# Diagnostic accuracy of oral swab for detection of pulmonary tuberculosis: a systematic review and meta-analysis

**DOI:** 10.3389/fmed.2023.1278716

**Published:** 2024-03-11

**Authors:** Fuzhen Zhang, Yilin Wang, Xuxia Zhang, Kewei Liu, Yuanyuan Shang, Wei Wang, Yuanyuan Liu, Liang Li, Yu Pang

**Affiliations:** ^1^Department of Epidemiology, School of Public Health, Cheeloo College of Medicine, Shandong University, Jinan, China; ^2^Department of Bacteriology and Immunology, Beijing Chest Hospital, Capital Medical University/Beijing Tuberculosis and Thoracic Tumor Research Institute, Beijing, China

**Keywords:** oral swab analysis, tuberculosis, diagnostic accuracy, systematic review, meta-analysis

## Abstract

**Objectives:**

Tuberculosis (TB) remains a significant concern in terms of public health, necessitating the timely and accurate diagnosis to impede its advancement. The utilization of oral swab analysis (OSA) presents a promising approach for diagnosing pulmonary TB by identifying *Mycobacterium tuberculosis* (MTB) within oral epithelial cells. Due to disparities in the diagnostic performance of OSA reported in the original studies, we conducted a meticulous meta-analysis to comprehensively assess the diagnostic efficacy of OSA in pulmonary TB.

**Methods:**

We conducted a comprehensive investigation across multiple databases, namely PubMed, Cochrane Library, Embase, Web of Science, ClinicalTrials.gov, Chinese BioMedical Literature Database (CBM), China National Knowledge Infrastructure Database (CNKI), and Wanfang China Science and Technology Journal Database to identify relevant studies. Out search query utilized the following keywords: oral swab, buccal swab, tongue swab, tuberculosis, and TB. Subsequently, we employed STATA 16.0 to compute the combined sensitivity, specificity, positive likelihood ratio, negative likelihood ratio, and diagnostic odds ratio for both the overall and subgroup analyses.

**Results:**

Our findings indicated that OSA has a combined sensitivity of 0.67 and specificity of 0.95 in individuals with pulmonary TB. Subgroup analysis further revealed that among adult individuals with pulmonary TB, the sensitivity and specificity of OSA were 0.73 and 0.93, respectively. In HIV-negative individuals with pulmonary TB, the sensitivity and specificity were 0.68 and 0.98, respectively. The performance of OSA in detecting pulmonary TB correlated with the bacteria load in sputum. Additionally, the sensitivity for diagnosing pulmonary TB using tongue specimens was higher (0.75, 95% CI: 0.65–0.83) compared to cheek specimens (0.52, 95% CI: 0.34–0.70), while both types of specimens demonstrated high specificity.

**Conclusions:**

To conclude, oral swabs serve as a promising alternative for diagnosing pulmonary TB, especially in adult patients. In addition, tongue swabs yield better sensitivity than cheek swabs to identify pulmonary TB patients.

**Systematic review registration:**

identifier: CRD42023421357.

## Introduction

Tuberculosis (TB), caused by *Mycobacterium tuberculosis* (MTB), remains a major significant contributor to mortality resulting from chronic infectious diseases, especially in immunodeficiency patients with HIV (1). The emergence of drug-resistant TB (DR-TB), encompassing extensively drug-resistant TB (XDR-TB) and pre-XDR-TB, has posed a substantial threat to TB control efforts in recent years (2). Timely and early diagnosis, along with prompt treatment, plays a crucial role in preventing the dissemination of drug-resistant TB (3). Currently, the majority of confirmed TB cases rely on the detection of MTB in sputum specimens, such as sputum smear, sputum culture, and MTB molecular in sputum (4–6). However, the reliance on sputum specimens presents limitations, particularly in pediatric patients and those who are unable to produce sputum (7). Additionally, the collection of sputum can cause potentially infectious aerosols that are harmful to healthcare workers and other patients (8). Moreover, the efficacy of TB diagnostic tests is contingent upon the quality of the sputum collected (9). Therefore, there is a pressing need for non-invasive alternatives to sputum-based testing that are easier, safer, and more efficient in diagnosing TB.

Several human samples, including exhaled breath condensate, saliva, urine, blood and stool, have been explored as alternatives to sputum for evaluating the diagnostic performance of TB (10, 11). Unfortunately, these alternative samples have demonstrated lower sensitivity or specificity than sputum. Recently, an oral (buccal) swab has emerged as a more encouraging alternative to sputum specimens for TB diagnosis. Previous studies have shown that oral swab analysis (OSA) can detect MTB DNA present in oral epithelial cells (12–15). OSA involves the utilization of a sterile brush to gently scrape cells from the dorsal surface of the tongue. This procedure is simple, quick, painless, non-invasive and does not generate aerosols, making it suitable for implementation in various healthcare settings, including tertiary hospitals, outpatient clinics, and community settings. Consequently, OSA holds particular value in identifying TB cases within diverse populations.

A series of studies examining the efficacy of OSA for diagnosing pulmonary TB have yielded inconsistent findings. For instance, one study conducted in South Africa evaluated swabs from adult subjects and found that OSA exhibited a sensitivity of 92.8% and specificity of 91.5% when compared to sputum GeneXpert testing (14). Similarly, another study involving 201 South African children with suspected pulmonary TB revealed that OSA demonstrated greater sensitivity than sputum testing in children who were negative for sputum, although its sensitivity was lower in sputum-positive children (16). By contrast, conflicting outcomes were noted in other studies, reporting lower sensitivity for detecting tubercle bacilli using oral swabs (13, 15, 17, 18). The limited sample sizes in previous studies undermine the confidence of these conclusions. Therefore, we conducted a meta-analysis to investigate the diagnostic value of OSA in detecting pulmonary TB while also conducting a comprehensive analysis of its diagnostic accuracy across different populations.

## Materials and methods

### Protocol registration

This systematic review and meta-analysis were conducted according to the Preferred Reporting Items for Systematic Reviews and meta-Analyses (PRISMA) statement. The protocol has been registered in PROSPERO (Number: CRD42023421357).

### Search strategy

We conducted a comprehensive and systematic search for relevant studies in electronic databases, including PubMed, Cochrane Library, Embase, Web of Science, ClinicalTrials.gov, Chinese BioMedical Literature Database (CBM), China National Knowledge Infrastructure Database (CNKI), and Wanfang China Science and Technology Journal Database, based on our review protocol. The most recent searches performed on 12 March 2023 used the following terms: (“oral swab” or “buccal swab” or “tongue swab”) and (“tuberculosis” or “TB”). No geographical or demographic restrictions were imposed during the search process, including the race or age of study participants. We considered studies published in both English and Chinese languages, and only included relevant data for our analysis.

### Eligibility criteria

We enrolled records that met the following eligibility criteria, which involved studies designed as diagnostic accuracy studies aiming to assess the diagnostic value of oral swabs for TB in human subjects. We exclusively considered studies that provided sufficient data for computing pooled sensitivity and specificity in human populations, including the count of true positives and negatives, as well as false positives and negatives. Only studies with complete data were included to avoid duplications. We excluded studies that lacked adequate data for calculating effect size or were missing other essential information. Moreover, publications encompassing reviews, case reports, abstracts, guidelines, and recommendations were also excluded, as they did not present primary results. Furthermore, studies lacking research indicators necessary for meta-analysis were also excluded.

### Data extraction

The study selection process was conducted independently by two investigators. In case of any discrepancies, a third author reviewed the articles, and a final consensus was reached through discussion. Initially, the investigators screened the literature based on titles and abstracts to exclude articles that did not meet the inclusion criteria. Subsequently, the remaining articles were re-evaluated by reading their full texts. Detailed information and data from the identified studies were extracted by the two investigators using a standardized data extraction form that had been pre-constructed. The following information was extracted for each study: the first author's name, year of publication, place of study, study population, population size, age, and sex distribution of participants, method of diagnosis, TB type, sensitivity, and specificity values with their corresponding 95% confidence intervals (CI), as well as the number of true positives, false positives, false negatives, and true negatives. The investigators extracted data according to various subgroups, including adults and children, TB patients with and without HIV and smear-positive and smear-negative TB. Furthermore, OSA samples collected from the tongue and cheek were evaluated in the diagnosis of TB.

### Statistical analysis

The quality of the included studies was evaluated using the widely Quality Assessment of Diagnostic Accuracy Studies-2 (QUADAS-2) tool (19). This tool assesses diagnostic accuracy based on four main points: selection of patients, index criteria, reference standard and flow and timing in the preliminary study. The quality rating scale is based on risk bias. Additionally, applicability concerns were taken into account when assessing the first three domains.

For the meta-analysis, STATA software version 16.0 was utilized. Heterogeneity was evaluated using both the chi-square and the *I*^2^ statistical tests. A significant level of heterogeneity was considered when *P* is greater or equal to 0.10, accompanied by an *I*^2^ values of 50% or higher. Acceptable inter-study heterogeneity was determined when the *I*^2^ value was equal to or < 50%. To mitigate the potential impact of the heterogeneity on the final conclusion, a random effects model was employed combine data from individual studies. This approach allowed for the estimation of sensitivity, specificity, positive/negative likelihood ratio and diagnostic odds ratio. The receiver operator characteristic (ROC) curve was utilized to obtain the area under the curve, providing a measure of the diagnostic accuracy. Publication bias was assessed using Deek's funnel plot methodology.

## Results

### Study characteristics

**Figure 1:**
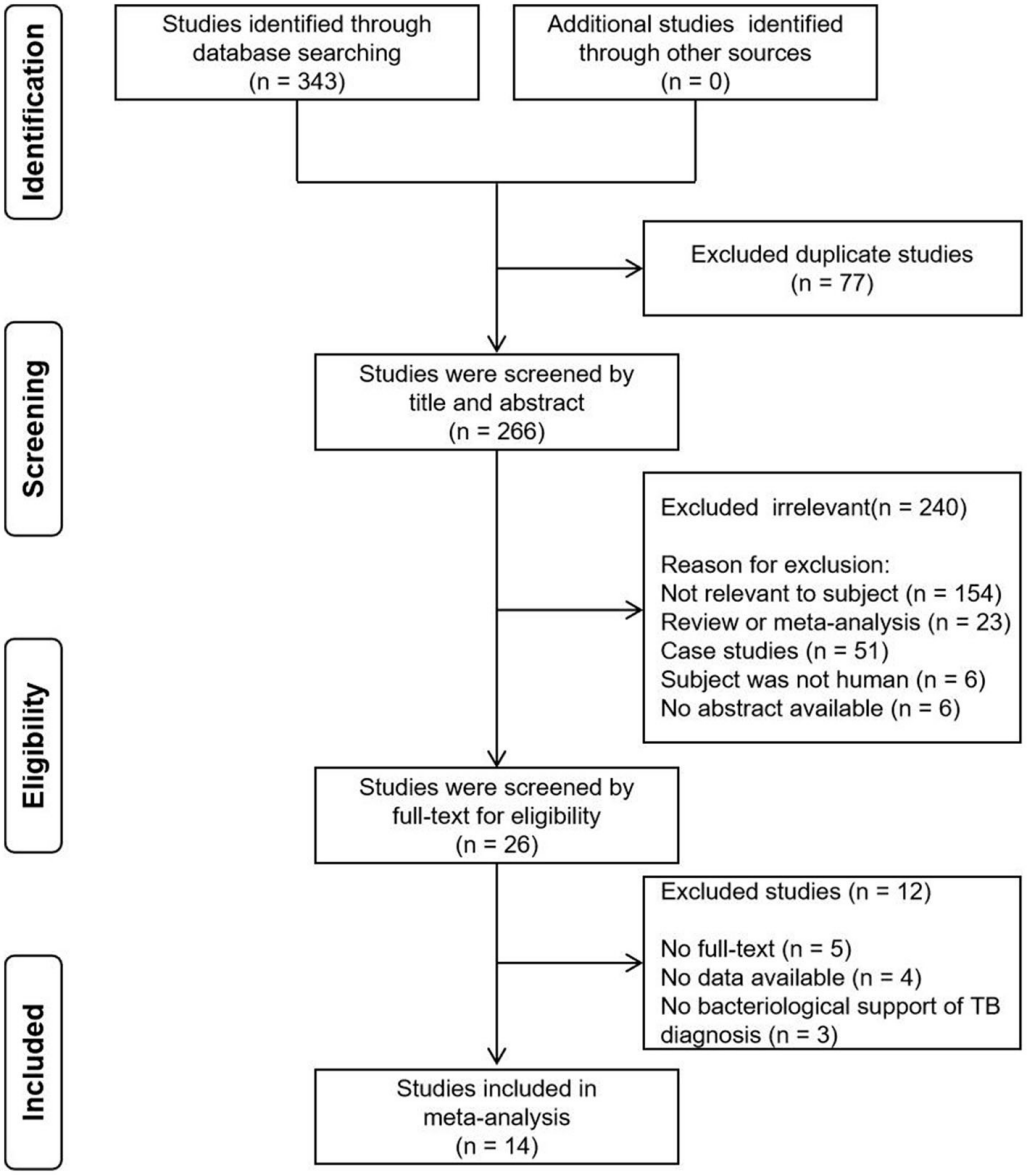
PRISMA flow diagram of the studies selection process.

Initially, a total of 343 records were identified, out of which 77 duplicates were removed. Subsequently, a screening process based on titles and abstracts was carried out, resulting in the exclusion of 240 irrelevant records. This narrowed down the selection to 26 articles, which were then assessed based on full-text contents. Among these, 12 articles were excluded due to reasons such as unavailability of full-text, absence of data and lack of bacteriological support of pulmonary TB diagnosis. Eventually, a systematic review was conducted, including 14 studies that fulfilled the inclusion criteria (Figure 1). Notably, two studies were included twice in separate records. In one study, TB was diagnosed using either culture or GeneXpert MTB/RIF as reference standards, resulting in two records being incorporated for this study (20). In the other study, two methods were employed in the OSA experiment. One used the double swab GeneXpert sample reagent method for GeneXpert MTB/RIF Ultra, while the other used the boil method for GeneXpert MTB/RIF Ultra, with two records also being included in this study (17). Among the remaining 16 records, two reported the diagnostic effectiveness of OSA in children with pulmonary TB, twelve explored the diagnostic effectiveness of OSA in adults with pulmonary TB, while the age range of the study population in two records could not be determined. Table 1 provides a concise summary of the key characteristics of the included studies. It is important to note that the diagnosis of pulmonary TB in all the included studies was based on confirmed cases using the reference standard.

**Table 1:** Baseline characteristics of OSA in the diagnosis of TB included in our analysis.

**References**	**Location**	**Study population**	**Sample size**	**Age, year**	**Sex (M/F)**	**No. of participants**				**Diagnostic method**	**Sensitivity (95% CI), %**	**Specificity (95% CI), %**	**TP**	**FP**	**FN**	**TN**
						**HIV positive**	**Confirmed TB**	**Suspected TB**	**Unlikely TB**							
Wood et al. (21)	South African; USA	Adult	40	NA	24/16	0	20		20	Xpert	90.0 (66.9–98.2)	100.0 (80.0–100.0)	18	0	2	20
Luabeya et al. (14)	South African	Adult	219	NA	NA	NA	148		71	Xpert or Culture	83.1 (70.6–91.1)	91.5 (81.9–96.5)	49	6	10	65
Nicol et al. (16)	South African	Children	165	2.5 (1.1–6.7)^a^	78/87	18	40	81	44	Xpert or Culture	42.5 (27.4–59.0)	93.2 (80.3–98.2)	17	3	23	41
Mesman et al. (15)	Peru	Adult	63	NA	NA	NA	33		30	Culture	45.5 (28.5–63.4)	100.0 (84.0–100.0)	15	0	18	26
Lima et al. (22)	Brazil	NA	256	NA	NA	NA	128		128	Xpert	51.6 (42.6–60.4)	100.0 (96.4–100.0)	66	0	62	128
Molina-Moya et al. (12)	Spain	Adult	266	48.8 ± 14.4^b^	157/109	NA	80	34	152	Xpert or Culture	36.3 (26.0–47.8)	79.6 (72.9–85.0)	29	38	51	148
Mesman et al. (23)	Peru	Adult	153	NA	NA	4	123		30	Culture	51.2 (42.1–60.3)	96.7 (80.9–99.8)	63	1	60	29
Flores et al. (13)	Peru	Children	288	NA	148/140	0	24	65	199	Culture	20.8 (7.9–42.7)	99.0 (96.0–99.8)	5	2	19	197
Wood et al. (20)^d^	Uganda; USA	Adult	194	NA	124/70	55	142		52	Xpert	88.0 (75.0–95.0)	79.2 (65.5–88.7)	44	11	6	42
Wood et al. (20)^e^	Uganda; USA	Adult	194	NA	124/70	55	142		52	Culture	91.5 (78.7–97.2)	66.1 (52.1–77.8)	43	19	4	37
Song et al. (24)	China	Adult	101	43.5 (17–88)^c^	69/32	0	46		55	Xpert and/or Culture	82.6 (71.7–93.6)	94.5 (88.5–100)	38	3	8	52
Shapiro et al. (25)	South African	Adult	131	36 (31–46)^a^	72/59	121	64		67	Xpert Ultra or Culture	65.7 (52.7–76.8)	77.6 (65.5–86.5)	42	15	22	52
Andama et al. (17)^f^	Uganda	Adult	183	33 (26–43)^a^	107/76	58	58		125	Xpert Ultra or Culture	72.4 (58.9–83.0)	100.0 (96.1–100.0)	42	0	16	119
Andama et al. (17)^g^	Uganda	Adult	183	33 (26–43)^a^	107/76	58	58		125	Xpert Ultra or Culture	77.1 (59.4–89.0)	100.0 (19.8–100.0)	27	0	8	2
Kang et al. (26)	Korea	Adult	272	58.8 ± 15.2^b^	174/98	1	99	29	144	Culture	64.6 (54.3–73.8)	86.1 (79.1–91.1)	64	20	35	124
LaCourse et al. (27)	Kenya	≥13 years	100	38 (30–44)^a^	52/48	54	20		80	Xpert or Culture	65.0 (40.9–83.7)	81.3 (70.6–88.8)	13	15	7	65

HIV, human immunodefificiency virus; Xpert, GeneXpert MTB/RIF; Xpert Ultra, GeneXpert MTB/RIF Ultra; IQR, interquartile range; SD, standard deviation; NA, not available applicable; TP, true positive; FP, false positive; FN, false negative; TN, true negative. ^a^Median ± IQR; ^b^mean ± SD; ^c^median ± range; ^d^TB Diagnosis using Xpert; ^e^TB Diagnosis using Culture; ^f^Xpert Ultra testing using the double swab GeneXpert sample reagent method; ^g^Xpert Ultra testing using the boil method.

### Risk of bias within studies

The 14 studies included in this research were assessed for risk of bias by using the QUADAS-2 tool. Among them, ten studies were found to have a low risk of selection bias, while 4 studies had an unclear risk of selection bias. Furthermore, eight studies exhibited a high risk of bias in the index test, as the interpretation of OSA results was influenced by prior knowledge of the reference standard results. In contrast, the reference standard and flow and timing displayed a low risk across all 14 studies. Lastly, two studies had unclear applicability concerns regarding patient selection. A detailed summary of the results can be found in Supplementary Table 1.

### Overall diagnostic accuracy of OSA in pulmonary TB

**Figure 2:**
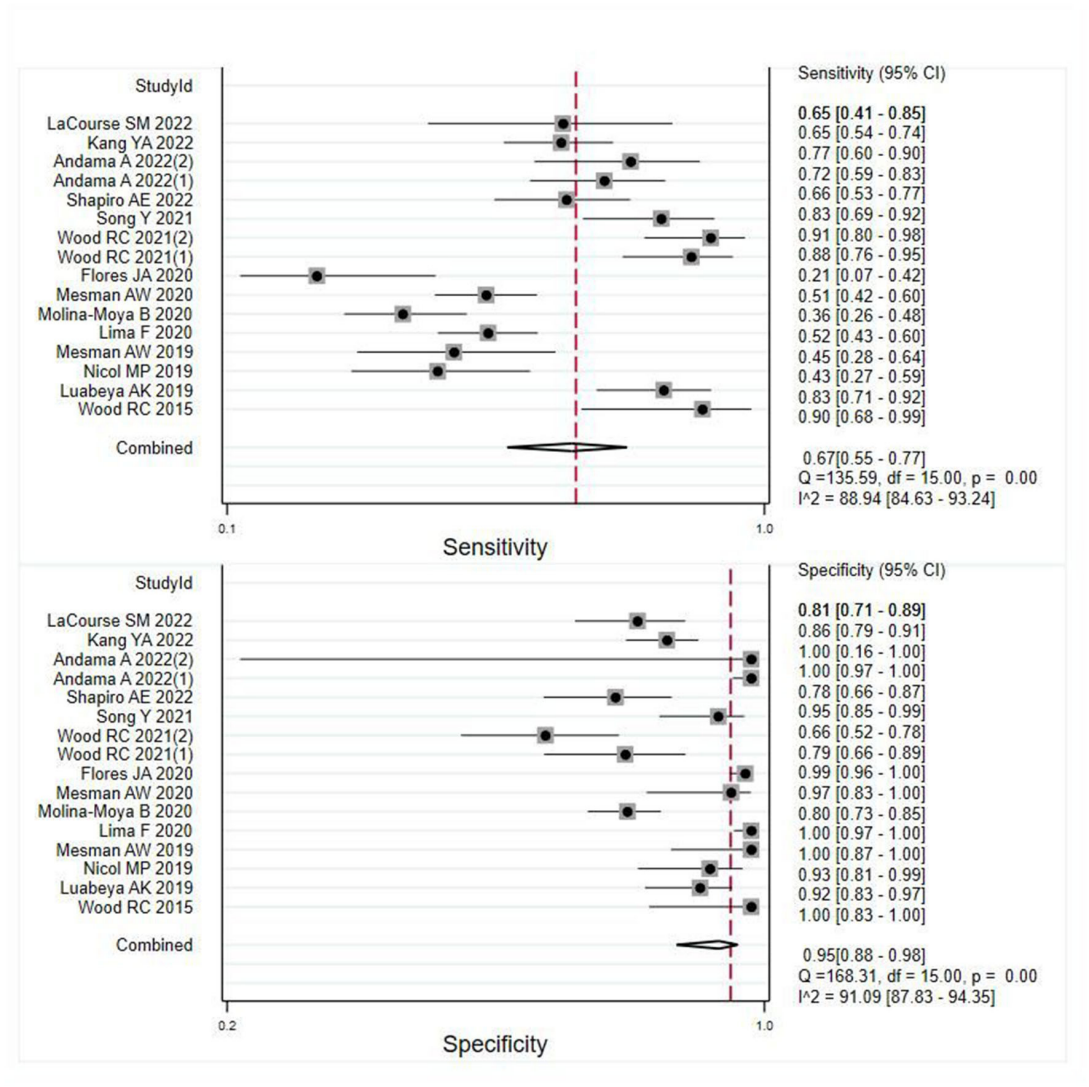
Meta-analysis results of the sensitivity and specificity of OSA in the diagnosis of individuals with pulmonary TB.

A total of 16 records were examined to assess the diagnostic value of OSA in pulmonary TB. Analysis of the results revealed that despite the heterogeneity resulting from variations in the operational processing of OSA among the studies (see Supplementary Table 3), OSA showed an overall pooled sensitivity of 0.67 (95% CI: 0.55–0.77, *I*^2^ = 88.94%) and an overall pooled specificity of 0.95 (95% CI: 0.88–0.96, *I*^2^ = 91.09%) in the diagnosis of pulmonary TB (Figure 2). Further subgroup analysis supported the overall findings, indicating a high specificity and diagnostic sensitivity of OSA in TB.

### Subgroups diagnostic accuracy of OSA in pulmonary TB

**Figure 3:**
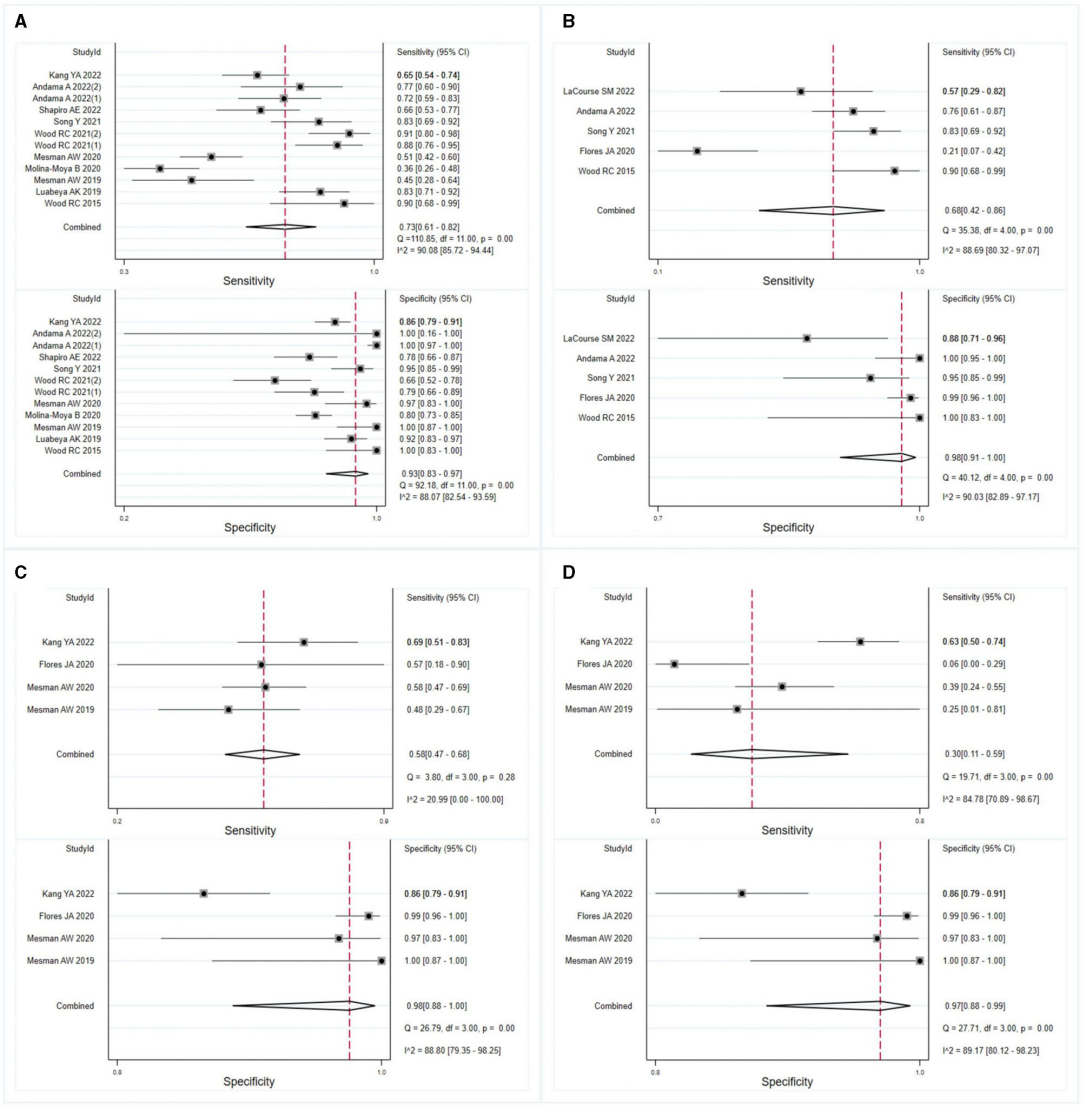
Meta-analysis assessed the sensitivity and specificity of OSA in diagnosing individuals with pulmonary TB. The results were stratified by **(A)** adult individuals with pulmonary TB, **(B)** HIV-negative individuals with pulmonary TB, **(C)** smear-positive individuals with pulmonary TB, and **(D)** smear-negative individuals with pulmonary TB.

**Figure 4:**
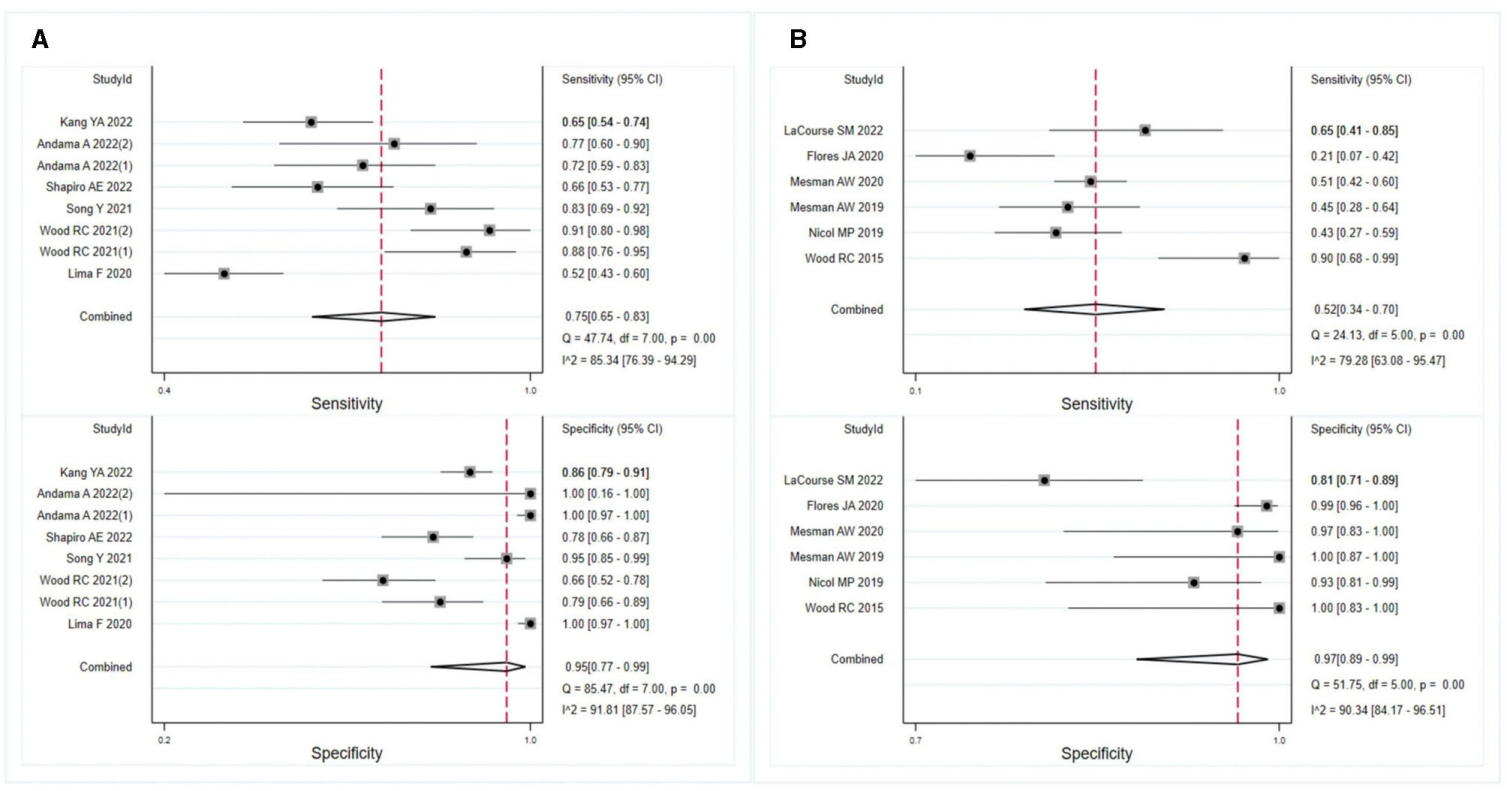
Meta-analysis of the sensitivity and specificity of tongue and cheek specimens of OSA in diagnosing pulmonary TB. **(A)** Results of meta-analysis on tongue specimens in diagnosing pulmonary TB; **(B)** results of meta-analysis on cheek specimens in diagnosing pulmonary TB.

The meta-analysis findings demonstrated that OSA exhibits sensitivity of 0.73 (95% CI: 0.61–0.82, *I*^2^ = 90.08%) and specificity of 0.93 (95% CI: 0.83–0.97, *I*^2^ = 88.07%) for diagnosing adults with pulmonary TB (Figure 3A). In HIV-negative individuals with pulmonary TB, the sensitivity and specificity of OSA were 0.68 (95% CI: 0.42–0.86, *I*^2^ = 88.69%) and 0.98 (95% CI: 0.91–1.00, *I*^2^ = 90.03%), respectively (Figure 3B). However, due to the limited number of studies available, a meta-analysis could not be conducted to evaluate the effectiveness of OSA in diagnosing children and HIV-positive individuals with pulmonary TB. Therefore, the diagnosis of OSA in these populations remains uncertain. Details are listed in Supplementary Table 2. Furthermore, for individuals with smear-positive pulmonary TB, OSA demonstrated a sensitivity and specificity of 0.58 (95% CI: 0.47–0.68, *I*^2^ = 20.99%) and 0.98 (95% CI: 0.88–1.00, *I*^2^ = 88.80%), respectively (Figure 3C). For smear-negative individuals with pulmonary TB, OSA exhibited a sensitivity and specificity of 0.30 (95% CI: 0.11–0.59, *I*^2^ = 84.78%) and 0.97 (95% CI: 0.88–0.99, *I*^2^ = 89.17%), respectively (Figure 3D). Moreover, we investigated the performance of OSA in diagnosing pulmonary TB using tongue and cheek specimens. The sensitivity of tongue and cheek specimens was 0.75 (95% CI: 0.65–0.83, *I*^2^ = 85.34%) and 0.52 (95% CI: 0.34–0.70, *I*^2^ = 79.28%), while the specificity was 0.95 (95% CI: 0.77–0.99, *I*^2^ = 91.81%) and 0.97 (95% CI: 0.89–0.99, *I*^2^ = 90.34%), respectively (Figure 4). Supplementary Table 2 displays all subgroup analyses of OSA in diagnosing pulmonary TB. Table 2 provides a summary of the meta-analysis results.

**Table 2:** Pooled results of OSA in the diagnosis of TB patients.

**Group**	**Sensitivity (95% CI)**	**Specificity (95% CI)**	**Positive LR (95% CI)**	**Negative LR (95% CI)**	**Diagnostic OR (95% CI)**	**SROC (95% CI)**
Overall	0.67 (0.55–0.77)	0.95 (0.88–0.98)	12.6 (5.4–29.0)	0.35 (0.26–0.48)	36 (14–89)	0.88 (0.85–0.91)
Adult	0.73 (0.61–0.82)	0.93 (0.83–0.97)	10.4 (4.2–25.7)	0.29 (0.20–0.43)	35 (12–102)	0.89 (0.86–0.92)
HIV negative	0.68 (0.42–0.86)	0.98 (0.91–1.00)	34.4 (7.3–162.5)	0.32 (0.15–0.67)	107 (17–690)	0.96 (0.94–0.98)
Smear positive TB	0.58 (0.47–0.68)	0.98 (0.88–1.00)	23.1 (5.0–107.0)	0.43 (0.34–0.54)	53 (12–244)	0.73 (0.69–0.76)
Smear negative TB	0.30 (0.11–0.59)	0.97 (0.88–0.99)	9.6 (3.5–26.5)	0.73 (0.52–1.01)	13 (5–38)	0.83 (0.79–0.86)
Tongue	0.75 (0.65–0.83)	0.95 (0.77–0.99)	13.8 (3.1–61.8)	0.26 (0.19–0.37)	53 (12–233)	0.87 (0.84–0.90)
Cheek	0.52 (0.34–0.70)	0.97 (0.89–0.99)	15.9 (4.6–54.8)	0.49 (0.34–0.72)	32 (8–124)	0.88 (0.85–0.91)

LR, likelihood ratio; OR, odds ratio; SROC, summary receiver operator characteristic curve; HIV, human immunodefificiency virus.

### Publication bias assessment

To assess publication bias, a funnel plot was employed (Supplementary Figure 1). The funnel plot clearly indicated the absence of significant publication bias in this meta-analysis (*P* = 0.99).

## Discussion

TB continues to pose a significant public health challenge, necessitating early detection and prompt treatment to curb its spread (3, 28). In recent years, researchers have developed oral swabs, as a non-invasive method for detecting MTB DNA, which can be obtained from the tongue, cheek or gums (14). While obtaining samples directly from the lower respiratory tract is of paramount importance and demonstrates superior efficacy in diagnosing TB, oral swabs present a more accessible alternative approach for TB diagnosis (17, 25). They have also been widely utilized in diagnosing TB in animals (29–31). Furthermore, the development of oral swabs aimed to mitigate the health risks associated with collecting sputum, which poses potential hazards for healthcare providers (32). In our meta-analysis, we comprehensively evaluated the diagnostic efficacy of oral swabs for pulmonary TB, providing novel insights into its potential utility. We observed that while the sensitivity of oral swabs for TB diagnosis was moderate, the specificity was notably high, yielding an overall favorable diagnostic effect. These findings suggest that oral swabs may serve as suitable specimens for rule-in testing in the diagnosis of pulmonary TB. Nonetheless, the records included exhibited considerable heterogeneity. This was mainly due to variations in the collection process of oral swabs and the number of participants in the studies. For instance, in a study by Wood et al. (21) found that oral swabs had high sensitivity and specificity for the diagnosis of pulmonary TB. However, the sample size was small, and the set positive threshold was larger than other studies (21). Another study by Wood et al. (20) revealed that increasing the number of participants and lowering the positive threshold of OSA resulted in a reduced specificity and slightly decreased sensitivity for diagnosing pulmonary TB. Nevertheless, the sensitivity still remained high in such instances, with Xpert-positive TB as the reference standard (20). Notably, when using the TB-LAMP method (24) or combining samples from two consecutive days (14) for OSA, both sensitivity and specificity were improved. These results highlight the presence of heterogeneity in the available data, emphasizing the need for further investigation into improving the implementation methodology of OSA and establishing appropriate thresholds to enhance sensitivity and specificity. Consequently, conducting more robust studies in this area becomes imperative.

In this study, we performed a subgroup analysis of various factors related to OSA for diagnosing individuals with pulmonary TB. Our finding revealed a high specificity of OSA for diagnosing pulmonary TB across various subgroups. Specifically, our analysis revealed a higher combined sensitivity of OSA for diagnosing pulmonary TB in adults compared to in children, as observed in studies by Nicol et al. (16) and Flores et al. (13). However, the sensitivity was lower for diagnosing pulmonary TB in HIV-negative individuals compared to HIV-positive individuals, according to LaCourse et al. (27), but higher than in the study by Andama et al. (17). These results suggest that OSA is a reliable method for diagnosing pulmonary TB in adults. However, caution is warranted in the case of HIV-positive individuals with pulmonary TB. Notably, Cox et al. found that the yield of microbiologic confirmation using oral swab specimens in children with pulmonary TB was suboptimal (18). Flores et al. demonstrated that tongue swabs can be employed for diagnosing TB in children who are clinically diagnosed with TB but are unable to produce sputum samples (33). Nevertheless, interpretation of these results requires caution as the number of studies on diagnosing OSA in children with pulmonary TB and HIV-positive individuals with pulmonary TB is limited. Further investigations are necessary to validate these findings. Moreover, in this meta-analysis, we assessed the diagnostic efficacy of OSA in individuals with pulmonary TB who had positive and negative sputum smear test results. Our analysis revealed that OSA exhibited a higher sensitivity in diagnosing pulmonary TB in individuals with positive sputum smear test results compared to those with negative test results. Flores et al. observed a positive correlation between positive sputum smear results and the detection of MTB in oral specimens (13). This suggests that the detection rate of MTB by oral swabs is proportional to the amount of MTB present in sputum. Consequently, oral swabs can serve as a viable substitute for sputum specimens in the diagnosis of TB.

While OSA can be utilized for diagnosing TB, it is important to note that their sensitivity is lower compared to the detection of TB using sputum specimens with the Xpert MTB/RIF and Xpert MTB/RIF Ultra methods, as indicated by previous study (34). However, in children with suspected pulmonary TB, OSA exhibits higher diagnostic sensitivity in comparison to using Xpert MTB/RIF for detecting sputum specimens and is comparable to the sensitivity of Xpert MTB/RIF Ultra for sputum specimen detection (35). Moreover, the sensitivity of OSA in diagnosing HIV-positive individuals with pulmonary TB (27) is similar to that of TB detection using Xpert MTB/RIF and Xpert MTB/RIF Ultra methods for sputum specimens (36). It is crucial to note that these conclusions are based on a limited number of studies examining the use of OSA as a diagnostic tool for pulmonary TB, highlighting the necessity for additional high-quality studies to confirm its utility in the future. To enhance the diagnostic effectiveness of OSA in pulmonary TB, optimization tests can be conducted. Supplementary Table 3 provides a comprehensive overview of the data we used from OSA in this study. Our previous research demonstrated that the TB-LAMP method we utilized to detect MTB in tongue swabs for diagnosing pulmonary TB yielded high sensitivity and specificity (24). Additionally, Andama et al. demonstrated that the sensitivity of Xpert MTB/RIF Ultra method for detecting MTB in tongue swabs was also relatively high, with a specificity of 100% (17). We recommend conducting further studies employing different methods to detect MTB in tongue swabs, thereby improving the sensitivity and specificity of OSA.

Tongue and cheek swabs are oral specimens commonly employed for the diagnosis of TB, although their exact diagnostic value remains uncertain. In this study, we conducted a meta-analysis focusing on tongue and cheek swabs collected via the use of OSA to diagnose pulmonary TB. The results of the meta-analysis revealed that tongue swabs exhibited higher sensitivity than cheek swabs when utilizing OSA for TB diagnosis, while their specificity remained comparable. The summary findings strongly underscore the importance of utilizing tongue swabs for the diagnosis of pulmonary TB, attributing their superior sensitivity to the fact that MTB primarily colonize the epithelial cells on the surface of the tongue. For future developments in the field of OSA-based diagnosis of pulmonary TB, we highly recommend the utilization of tongue swabs. However, it is crucial to note that despite conducting a subgroup analysis, the presence of substantial heterogeneity observed amongst the results emphasizes significant variability across the studies included in the analysis. Hence, there is a pressing need to establish standardized protocols for conducting OSA to ensure consistency and reliability in its diagnostic applications.

A limitation of the current work is the relatively small number of publications available on this particular topic. Despite ongoing and extensive research in the field of OSA for diagnosing TB, only a limited number of articles met our eligibility criteria. Adding complexity to the analysis is the lack of methodological consistency across studies and insufficient reporting of methods and outcome parameters. Additionally, there are several other confounding factors that might have affected the diagnostic effectiveness of OSA in pulmonary TB, including the method of sample collection, swab storage buffer, storage period before testing, DNA extraction method and detection method. Consequently, the heterogeneity among the included studies was significant, thereby impacting the analysis in this study.

In conclusion, our findings suggest that oral swabs serve as a promising alternative for diagnosing pulmonary TB, particularly in adult patients. Notably, tongue swabs exhibit superior sensitivity compared to cheek swabs in detecting individuals with pulmonary TB. However, further rigorous assessment with a larger sample size, focusing on populations such as HIV-positive individuals and children, is warranted to validate the diagnostic performance of OSA. Furthermore, we strongly advocate for future studies to offer comprehensive information regarding collection procedures, enabling the field to better comprehend the necessary trade-offs required for the successful implementation and scalability of OSA in TB diagnosis.

## Data availability statement

The original contributions presented in the study are included in the article/Supplementary material, further inquiries can be directed to the corresponding authors.

## Author contributions

FZ: Writing – original draft, Data curation, Formal analysis, Investigation, Methodology, Software. YW: Data curation, Formal analysis, Investigation, Validation, Writing – review & editing. XZ: Data curation, Formal analysis, Investigation, Writing – review & editing, Supervision. KL: Formal analysis, Supervision, Writing – review & editing, Methodology. YS: Formal analysis, Methodology, Supervision, Writing – review & editing. WW: Formal analysis, Methodology, Supervision, Writing – review & editing. YL: Formal analysis, Methodology, Supervision, Writing – review & editing. LL: Conceptualization, Funding acquisition, Writing – original draft. YP: Conceptualization, Funding acquisition, Writing – original draft.
